# Preservice and Inservice Teachers' Noticing of Explicit Instruction for Self-Regulated Learning Strategies

**DOI:** 10.3389/fpsyg.2021.630197

**Published:** 2021-03-26

**Authors:** Tova Michalsky

**Affiliations:** School of Education, Bar-Ilan University, Ramat Gan, Israel

**Keywords:** self-regulated learning strategies, noticing, preservice teachers, inservice teachers, videotaped lesson analysis

## Abstract

Contemporary theories of learning and instruction as well as a large body of research have pinpointed the benefits of effective self-regulated learning (SRL) for students' academic achievements, yet research findings indicate that teachers' actual promotion of students' SRL strategies and students' actual use of such strategies are less common than expected. To extend the investigation of how and when teachers' expertise develops regarding SRL instruction practices in authentic classrooms, the current study compared preservice vs. inservice teachers' “noticing” of explicit SRL teaching behaviors in videotaped classroom vignettes. Preservice teachers in a university teacher training program (*N* = 296) and inservice elementary, junior high, and high school teachers (*N* = 305) were presented with six online video cases accompanied by questions about the videotaped teachers' instruction of SRL planning, monitoring, and evaluation strategies. The results suggested that, overall, both preservice and inservice teachers failed to notice the expert teachers' explicit SRL teaching. Furthermore, their noticing ability failed to increase over the career span, with growing teaching experience. Thus, targeted instruction is recommended during both preservice training and inservice development programs to promote all teachers' application of evidence-based explicit SRL teaching strategies.

## Introduction

Our complex, rapidly changing world, with its overabundance of information, stimuli, and demands, creates a growing need for self-initiated and self-managed learning. Knowing how to manage one's own learning activities has become, in short, an important survival tool (Bjork et al., 2013). Moreover, the self-management of one's learning or one's self-regulated learning (SRL) is a central feature of most contemporary theories of learning and instruction in the educational system. Findings from research based on SRL theories have provided evidence that the explicit teaching of SRL strategies to students—such as planning, monitoring, and evaluating their own task performance—can have a significant positive impact on their academic achievements (e.g., Mirhosseini et al., 2018; Xiao et al., 2019; Michalsky, 2020a).

Nevertheless, research has suggested that such explicit SRL strategy teaching is often lacking in many school and university classrooms (Dignath and Veenman, 2020). Why is more widespread instruction not occurring in school classrooms today, which utilizes evidence-based knowledge to implement effective SRL teaching strategies? Contemporary educational experts and policymakers expect teachers to know how to teach effective SRL strategies to their students, but perhaps teachers may not possess the necessary meta-knowledge to apply the specific learning strategies that may optimize students' SRL acquisition because classrooms comprise contextually rich teaching situations that are often “messy” in terms of their underlying logical structures (Soodla et al., 2017; Zohar and Lustov, 2018).

This study aimed to investigate teachers' noticing—their “ability to attend intentionally to classroom events that are important to the processes of teaching and learning, for example, events that influence student learning in a positive or negative way” (Schäfer and Seidel, 2015, p. 37)—with regard to SRL teaching strategies. Researchers have proposed that a key component of teaching is the ability to notice and interpret what happens in the classroom. Classrooms are complex environments with many events unfolding simultaneously; thus, interpreting important features of classrooms requires skill. This ability has been called teachers' professional vision (Goodwin and Schroeder, 1994), which is described as the teachers' ability to “make sense of what is happening in their classrooms” (Sherin, 2007, p. 384). From the perspective of education reform policies promoting “meaningful learning,” the development of teachers' in-the-moment professional vision with respect to student thinking is necessary for teachers to adopt a responsive or student-centered teaching approach (Kersting et al., 2002). Nevertheless, research on professional development has also pinpointed the high correlation between teachers' noticing of meaningful teaching processes in observed lessons and those teachers' actual teaching of those processes in practice (Schäfer and Seidel, 2015; Pielmeier et al., 2018). Thus, teachers' noticing predicts their practical teaching (Louie, 2018).

In the SRL context, researchers have asserted that teachers' development of skills for noticing SRL will lead to real-time teaching of SRL processes (Michalsky, 2014, 2020a; Kramarski and Kohen, 2017). Well-developed noticing skills can direct teachers' attention to relevant SRL-promoting affordances that are embedded in specific teaching contexts. Moreover, teachers' noticing of explicit SRL strategy teaching may enhance transfer by enabling identification of the deeper shared characteristics and logic of various SRL-promoting learning situations that appear to differ in their surface affordances (Brown et al., 1983; Veenman, 2011; Yanqun, 2019).

Exploring the notion that perhaps teachers today often do not teach SRL strategies to their students because the teachers themselves may not possess adequate knowledge about how to teach those strategies, the current study focused on examining the ability of teachers to notice and identify real-time implementation of explicit SRL teaching strategies in authentic classrooms. In addition, the current study focused on whether teachers' noticing expertise about SRL teaching develops with experience across the teaching career span in order to determine where and when future interventions might be most needed for preservice and inservice teachers.

### Self-Regulated Learning

Researchers and scholars have proposed many different SRL models and constructs, but they do share some basic assumptions about learning and regulation (Butler and Winne, 1995; Pintrich, 2000; Zimmerman, 2000, 2008; Schraw et al., 2006). Specifically, SRL is generally considered to be an active process referring to “self-generated thoughts, feelings, and actions that are planned and cyclically adapted to the attainment of personal goals” (Zimmerman, 2000, p. 14). Zimmerman's (2000; 2008) well-accepted SRL model thus emphasizes three recursive phases during learning—forethought, action and performance, and evaluation—which are implemented within an environmental context. Corresponding to these three phases are three desired SRL strategies—planning, monitoring, and evaluation, respectively—which have been identified as effective and robust. Research has demonstrated that teachers can succeed in developing these SRL strategies in their students *via* direct instruction of these SRL strategies in their classrooms (Zimmerman, 2002; Greene and Azevedo, 2009), as will be detailed next.

### Direct, Explicit Instruction of SRL Strategies

Direct SRL instruction involves explicitly explaining different SRL strategies to students as well as how those strategies are used and what skills are involved in using those strategies (Zimmerman, 2008). The focus of this kind of direct instruction is on modeling and demonstration. When teachers model and explain their own thought processes necessary for completing activities and assignments, students are more apt to understand and begin to use those same processes on their own (Boekaerts and Corno, 2005; Dignath and Veenman, 2020). Research has shown that this type of explicit instruction can be the best initial strategy for encouraging students to be more self-regulative (Levy, 1996; Dignath and Büttner, 2018).

Good strategy instructors must know not only which SRL strategies are effective for learning but also how to teach them by embedding strategy instruction into content teaching. Embedment can be implemented by strategy instructors if they (a) introduce the strategy by modeling it and describing it, (b) sell the strategy by telling why it works, (c) generalize the strategy by telling where else it is useful, and (d) perfect the strategy by providing practice opportunities (Pressley and Woloshyn, 1995; Kiewra, 2002). Giving students explicit directions for using SRL strategies (planning, monitoring, and evaluation) should include when to use them, what goals to set, how to pursue those goals, how to monitor strategies and movement toward goal achievement as well as explicit information about the strategies' meaning and importance, which may offer meta-knowledge that can lead to a future transfer of the learned strategies. Direct explicit SRL strategy instruction contrasts with teachers' mere modeling of a strategy's use and verbalization of thought processes, which can implicitly induce students to show certain behaviors but does not inform students about the activity's significance (Dignath and Veenman, 2020).

Explicit SRL teaching differs according to the SRL phase of learning, thereby requiring different teacher behaviors to elicit students' skills for planning, monitoring, and evaluation. Examples of explicit teaching behaviors to instruct learners to use SRL strategies related to planning may include teachers' modeling of how to generate self-directed questions as they begin to work on an academic task: “What is the goal of this task? What strategies should I use? And why? Do I understand what I need to do?” (Veenman et al., 2006). Examples of explicit teaching behaviors to instruct learners to use self-observation SRL strategies related to monitoring may include teachers' modeling of how to generate self-directed questions during their work on an academic task: “Am I going in the right direction? Am I working according to my plan? Are the strategies that I am using helpful?” (Michalsky and Kramarski, 2015). Examples of explicit teaching behaviors to instruct learners to use SRL strategies related to evaluation may include teachers' modeling of how to generate self-directed questions at the end of the process of working on an academic task: “Does the solution make sense? Am I satisfied with the way I handled the task? What can I do better or change in my next academic task?” (Dignath and Veenman, 2020).

### Teachers' Development of Noticing Skills

Noticing is a subcomponent of the term “professional vision” that describes teachers' ability to perceive (to notice) and then to make sense of (to describe, explain, interpret, review, and predict) relevant classroom situations in order to consistently improve their teaching (Sherin, 2007). Though definitions of professional vision differ to some degree, they all include both noticing processes and knowledge-based reasoning processes as two main subcomponents (Blomberg et al., 2011; Sherin et al., 2011; Steffensky et al., 2015).

The current study explores only the first stage of professional vision—the noticing stage. Noticing is considered to be a crucial component of teacher competence because teachers must first pay attention to and identify the targeted or important teaching/learning events—must notice them—while ignoring the untargeted or unimportant events that are occurring in the same observed classroom sequence (König et al., 2014) in order to proceed to the next stages of professional vision. Only after they notice and pinpoint relevant events can teachers interpret them and reason about which particular aspects may foster or constrain students' learning based on teachers' existing professional knowledge (Hammerness et al., 2002; Borko et al., 2008; Blomberg et al., 2011; König et al., 2014; Seidel and Stürmer, 2014). Thus, professional vision helps teachers connect theory with practice by applying their conceptual knowledge of teaching/learning to authentic classroom situations (Kersting et al., 2010; König et al., 2014). For example, Kersting et al. (2010) found that teachers' mathematical pedagogical knowledge correlated highly with their ability to analyze videotaped mathematics lessons.

Blömeke et al. (2015) conceptualized the situation-specific skills within professional vision as mediators between professional knowledge and actual classroom practice, that is, in the competence model developed by Blömeke et al., situation-specific skills like noticing in an observed classroom scenario are predictors of actual teaching ability. Importantly, research has indicated that teachers' professional vision affects real-time instructional quality and student learning (Kersting et al., 2012; van Es et al., 2017). For example, Gibson and Ross (2016) found that a professional development intervention to promote inservice teachers' professional vision regarding instruction of reading comprehension significantly increased their real-time teaching of reading comprehension.

How do noticing skills develop over teachers' career trajectory? In domains not directly related to SRL teaching, expertise research (for an overview, see Berliner, 2001) as well as recent career-stage studies on situation-specific skills such as classroom management and students' cognitive dissonance have shown that inservice teachers are better able to notice various classroom situations than novice teachers (Seidel et al., 2007; König and Kramer, 2016; Meschede et al., 2017). For example, studies unrelated to SRL found that preservice teachers tended to focus on superficial aspects of classroom situations, whereas inservice teachers were able to quickly identify meaningful relevant aspects and draw conclusions for subsequent actions (Sabers et al., 1991; Wolff et al., 2016). Blömeke et al. (2016) likewise showed that teachers' skills for noticing lesson goals, classroom climate, and students' content knowledge misconceptions were better among later-career teachers than among early-career teachers. However, examining only early-career teachers, König et al. (2015) found that noticing of classroom situations was not predicted by teachers' amount of practical teaching experience, suggesting that noticing skills may take lengthy durations over years to develop (Kersting et al., 2012).

To promote teachers' noticing of SRL in observed classrooms, Michalsky (2020a,b) recently reported on two interventions' outcomes, but only for preservice teachers. In one study, prompts that merely hinted to preservice teachers “what” strategy they should notice in a videotaped classroom SRL teaching event (planning, monitoring, or evaluation) were found to be more effective than direct prompts pinpointing the time stamp for “when” to notice an SRL teaching event without specifying which strategy (Michalsky, 2020a). In the other study, the promotion of dual learning from both teacher and student perspectives in authentic videotaped classrooms was more effective for developing preservice teachers' noticing of SRL teaching events (on planning, monitoring, or evaluation strategies) than learning from only one perspective (Michalsky, 2020b).

In sum, there is a paucity of research specifically comparing early- vs. late-career teachers' noticing skills in the domain of authentic SRL instruction aiming to promote the three learning strategies—planning, monitoring, and evaluation—that have repeatedly been linked to students' SRL and to their academic achievements in all contents and across ages (Seidel and Shavelson, 2007; Carter, 2009; Tennant, 2019). In view of accumulating evidence that direct SRL strategy instruction is only infrequently taking place in today's classrooms (e.g., Organisation for Economic Co-operation Development, 2017; Dignath and Veenman, 2020), such career-span research is much needed to better understand how and when teachers may develop basic professional vision capacities for effective SRL teaching.

### The Current Study

This study aimed to extend a prior empirical investigation of teachers' noticing skills—as the first stage in professional vision—to the under-investigated domain of explicit SRL teaching to promote students' planning, monitoring, and evaluation strategies. The study also aimed to conduct a novel comparison of preservice vs. inservice teachers with regard to noticing of explicit SRL instruction in authentic classrooms and to examine the extent to which inservice teachers' SRL teaching noticing skills develop with additional teaching experience. Thus, hypotheses were formulated to address two research questions:

1. Do preservice and inservice teachers differ in their skills for noticing videotaped teachers' explicit SRL strategy teaching (for planning, monitoring, and evaluation strategies)? In line with prior research showing that inservice teachers are better able to notice general pedagogical aspects of classroom situations (e.g., learning goals, class climate, student misconceptions) compared to novice teachers (e.g., Berliner, 2001; Seidel et al., 2007; Blömeke et al., 2016; Todorova et al., 2017), the current inservice teachers were hypothesized to outperform the preservice teachers in noticing videotaped teachers' explicit instruction for the planning, monitoring, and evaluation SRL strategies.

Inasmuch as the current investigation extended to a previously unexplored area, the analysis for the first research question also examined the possible effects of demographic and setting characteristics: participants' age, sex, and school type for the inservice teachers. Age differences may be expected in correlation with increasing teaching experience (Graham et al., 2020), although prior research on teachers' professional vision skills and SRL skills did not yield significant sex differences among teachers (Seidel et al., 2011; Seidel and Stürmer, 2014). Regarding the possible relevance of school type for inservice teachers' noticing skills, observations of primary vs. secondary school teachers in the classroom showed no significant differences between the two groups in their actual explicit instruction of SRL strategies (Dignath and Büttner, 2018). To be noted is Dignath and Büttner's study which examined actual behavior rather than noticing skills, which have not yet been adequately investigated. The additional interviews that they conducted with the high school teachers revealed that although “most of them valued cognitive, metacognitive, and motivational components of SRL,” the “teachers lacked pedagogical knowledge about SRL teaching and were rather reluctant to promote it” (p. 127).

2. Does noticing expertise about explicit SRL strategy teaching develop with growing experience across the teaching career span among the inservice teachers? In line with prior research indicating that teachers' content knowledge, pedagogical knowledge, and ability to teach learning skills develop across their career (e.g., Blömeke et al., 2016; Myrberg et al., 2019), the current inservice sample was hypothesized to demonstrate growth in their noticing skills coinciding with their years of teaching experience.

## Materials and Methods

### Participants and Procedures

The participants were 601 Israeli teachers, comprising 296 preservice teachers enrolled in a university teacher training program in central Israel (ages 22–36 years, *M* = 26.46, *SD* = 4.51; 75.2% females) and 305 active inservice teachers (ages 26–56 years, *M* = 42.81, *SD* = 8.33; 78.3% females) in a range of teaching settings. The preservice teachers completed the online study measures individually as a class demonstration at the beginning of the first lesson of a university course, *Learning and Teaching Methods*, in the second year of their teaching certification studies. Inservice teachers, who were recruited as a convenience sample using the snowball method, completed the online study measures individually following an invitation that was posted on online teacher discussion forums or distributed by several teachers among their colleagues. Only active inservice teachers were included in the inservice group. According to self-reports, the inservice teachers taught a diverse range of subjects in public elementary schools (36%), junior high schools (23%), and high schools (37%) or in private schools (4%). The self-reported teaching experience for inservice teachers ranged from 1 to 32 years (*M* = 16.35 years, *SD* = 4.17).

This study was carried out in accordance with the recommendations of the university's institutional review board, the departmental ethics committee, and the ethical principles of the American Psychological Association. Approval by an ethics committee was not required according to the institutional and departmental guidelines. The participants completed the online study measures anonymously and voluntarily. They were informed that the data were being collected for research purposes by the author. For inservice teachers, consent was implied by completion of the online measures. The preservice teachers were asked by the university course instructor to indicate online whether they consented for their responses to the class demonstration to be utilized for the purpose of the study, and three university students who did not consent were excluded from all analyses.

### Measures

All 601 participants completed two online measures: a brief demographic questionnaire and the “Observer” instrument developed for the purpose of the present study. The online Observer assessed the participants' noticing of the SRL teaching behaviors that were explicitly demonstrated by videotaped teachers during authentic classroom situations (Michalsky, 2014; Seidel and Stürmer, 2014).

#### Observer Instrument Development

The Observer stimuli comprised six short video clips (2–4 min each) depicting diverse classroom teaching scenarios, taken from the Ministry of Education's video stockpile of middle school expert and non-expert teachers collected as part of the Third International Programme for International Student Assessment (PISA) Video 2013 Study (Centre for Educational Technology, 2014). To develop stimuli for the Observer instrument in which the targeted SRL teaching behaviors would be either clearly present or clearly absent, a team of three judges initially classified a pool of 26 clips. Each clip was coded for the explicitness of the videotaped teacher's instruction of all three SRL strategies (planning, monitoring, and evaluation) on a seven-point Likert scale ranging from highly implicit/none (1) to highly explicit (7).

The judges were a university professor with expertise in teacher education research and two independent research experts in the field of teaching and learning, who were not part of the research team. All three judges had at least 5–10 years of experience in teacher education and had 100–400 h of experience in systematic observation of classroom situations according to the SRL components under investigation. A mean Cohen's kappa (κ) of 0.79 across the three judges indicated a satisfactory level of inter-judge consistency (Seidel et al., 2010). In cases where the experts disagreed, agreement was reached by consensus validation.

Of the initial pool, six clips were selected as the stimuli for the current Observer instrument. The judges classified three of these six selected videotaped teachers' SRL teaching events as highly explicit (rated 7) in the targeted SRL strategy, thus identifying one clip each that demonstrated “expert” explicit teaching of planning (forethought), monitoring (action/performance), or evaluation (Seidel et al., 2011; Stürmer et al., 2013). The judges likewise classified three of these six videotaped teachers' SRL teaching events as highly implicit or non-existent (rated 1) in the targeted SRL strategy, thus identifying one clip each that demonstrated “non-expert” implicit/no teaching of planning, monitoring, or evaluation. To be noted is the fact that each of the six clips included explicit teaching of more than one strategy to reflect authentic, “messy,” contextually rich classrooms (Soodla et al., 2017; Zohar and Lustov, 2018). However, the participants were asked about only one strategy per clip (e.g., one clip asking the participants about monitoring might also include moderately explicit SRL teaching of planning).

Thus, the Observer stimuli comprised three pairs of video clips, with one pair each to assess the participants' noticing of videotaped teacher behaviors that explicitly taught planning, monitoring, and evaluation SRL strategies. In each pair, the expert vignette provided modeling of “good,” explicitly demonstrated teaching of that SRL strategy, whereas the non-expert vignette exemplified merely implicit or no SRL strategy teaching. For example, in the pair of clips referring to teaching of the SRL monitoring strategy, the expert teacher's clip showed Gila explicitly instructing her students: “During your work on your learning task, are you working according to your advance plan? Are the strategies that you chose suitable to your learning task?” Gila asked these questions when introducing her students to a learning task—a text to read accompanied by related questions on their lesson's subject, designed to correspond with the Israeli PISA conceptual framework for literacy (Organisation for Economic Co-operation Development, 2014, 2017). In contrast, in the non-expert clip asking the study participants about teaching of the SRL monitoring strategy, Tali merely told her students to “stop and check yourselves” when introducing the learning task.

#### Observer Instrument Administration Procedure

The online administration of the three-item Observer instrument to the participants comprised the presentation of each pair of video clips (one expert and one non-expert, in counterbalanced order across participants) followed by a single question referring to the SRL strategy instruction to be noticed in that pair of clips, as seen in the Appendix. The question asked the preservice and inservice teachers to rate the extent to which one of the two observed videotaped teachers had taught the relevant strategy (planning, monitoring, or evaluation) more explicitly than the other along a seven-point scale (1–7). As seen in the Appendix, to prevent straightlining responses, for the first item (planning), the expert teacher (with the pseudonym David) was on the left of the Likert scale and the non-expert (Natalie) was on the right, whereas for the second and third items (monitoring and evaluation, respectively), the non-experts (Tali, Sara) appeared on the left and the experts (Gila, Shelli) were on the right. The participants could return to the previous video clips and questions. The data log showed that 294 of the 377 participants who returned to prior clips and questions (78%) retained their original answers. The online Observer instrument took ~30 min to complete.

### Data Analysis

On each of the three questions, a rating of 4 represented a neutral score, reflecting that the participant assessed the two videotaped teachers in the pair as similar in their explicitness of SRL strategy teaching. Endorsement of one over the other teacher in a pair was indicated by a rating of 1–3 given to teachers presented on the left side of the Likert scale or by a rating of 5–7 given to teachers presented on the right side. Expert and non-expert teachers were scored differently. For experts, scoring was reversed for David such that a final score of 5–7 indicated correct noticing of David, Gila, and Shelli's highly explicit teaching (of planning, monitoring, and evaluation strategies, respectively), whereas a final score of 1–3 indicated poor noticing of their expert SRL teaching. For non-experts, scoring was reversed for Tali and Sara such that a final score of 5–7 indicated correct ratings (low noticing) of Natalie, Tali, and Sara's highly implicit teaching (of planning, monitoring, and evaluation strategies, respectively), whereas a final score of 1–3 indicated incorrect attribution of explicit teaching to the non-expert teachers. A mean combined score was computed for all three strategies together to reflect each participant's overall accuracy of noticing about explicit SRL strategy teaching.

To examine possible response bias and ensure data normality, preliminary analyses were conducted to check the responses' deviation from the neutral score and the data's distribution, kurtosis, and asymmetry. To examine the participants' level of noticing skills in each group separately (preservice and inservice) and in comparing the two groups, the final scores were each compared with the neutral (4) response using *t*-tests, and Cohen's *d* was calculated as the ratio between the [mean score minus the neutral score (4)] and [the standard deviation]. Analysis of covariance (ANCOVA) was conducted to examine demographic and setting characteristics. To determine the extent to which years of experience correlated with inservice teachers' noticing skills, Spearman's rank-order correlations were conducted.

## Results

Table 1 presents the descriptive and inferential statistics for the participants' noticing scores by SRL strategy type (planning, monitoring, and evaluation) and by group (preservice and inservice). Preliminary analysis was conducted to check the responses' deviation from the neutral score. As seen from Table 1, the *t-*tests showed that, in the preservice teacher group, the mean scores differed significantly from a neutral response (score of 4) when noticing the videotaped teachers' explicit instruction of monitoring and evaluation strategies and for the combined Observer score, but the mean score for noticing planning strategy instruction did not significantly differ from the neutral score. The inservice teachers' mean scores differed significantly from the neutral score in noticing the videotaped teachers' explicit instruction of the evaluation strategy and for the combined Observer score, but their mean scores for noticing explicit instruction of the planning and monitoring strategies did not significantly differ from the neutral score.

**Table 1:** Descriptive and inferential statistics for accurate noticing of videotaped teachers' explicit instruction of self-regulated learning (SRL), by strategy type and group.

**Noticing of explicit SRL strategy teaching**	**Teacher group**												**Groups' comparison (*n* = 601)**		
	**Preservice (*n* = 296)**						**Inservice (*n* = 305)**								
	**% correct noticing**	**Score from 1 to 7**		***M*** **vs. neutral (rating of 4)**			**% correct noticing**	**Score from 1 to 7**		***M*** **vs. neutral (rating of 4)**			***M*** **vs. neutral (rating of 4)**		
		***M***	***SD***	***t* (295)**	***p***	**Cohen's *d***		***M***	***SD***	***t* (304)**	***p***	**Cohen's *d***	***t* (599)**	***p***	**Cohen's *d***
Planning: David > Natalie	41	3.12	2.14	0.51	0.721	0.06	39	3.15	2.26	0.53	0.602	0.07	0.09	0.996	0.01
Monitoring: Gila > Tali	21	3.02	2.16	4.23	0.000	0.46	33	3.87	2.19	0.32	0.841	0.04	3.01	0.018	0.43
Evaluation: Shelli > Sara	25	2.69	2.54	6.14	0.000	0.61	14	2.28	1.75	9.23	0.000	0.89	1.36	0.186	0.32
Combined score		2.94	1.17	5.49	<0.001	1.36		3.12	1.31	4.63	<0.001	1.12	0.61	0.497	0.12

*The expert teachers were David, Gila, and Shelli. The non-experts were Natalie, Tali, and Sara*.

Table 2 presents the distribution of responses (in percentages) by group and SRL strategy. As seen on the table, the distribution of preservice and inservice teachers' responses was similar for their noticing of explicitly taught planning and evaluation strategies but differed for their noticing of monitoring strategy teaching. The latter strategy elicited the highest percentage of neutral responses (4 on the Likert scale, indicating uncertainty) in both groups, although the percentage of inservice teachers who noticed explicit teaching of monitoring was significantly higher than that of preservice teachers.

**Table 2:** Teachers' distribution of observer instrument responses (in percentages) by group.

**Self-regulated learning strategy question (expert > non-expert clip)**	**Group**	**Responses on seven-point scale (after reversing)**						
		**Endorsement of implicit strategy teaching**						**Endorsement of explicit strategy teaching**
		**1**	**2**	**3**	**4**	**5**	**6**	**7**
Planning: David > Natalie	Preservice	24.92	11.60	5.02	2.00	10.43	20.34	25.69
	Inservice	23.83	12.63	2.88	7.10	14.20	20.29	19.07
Monitoring: Gila > Tali	Preservice	34.83	17.76	16.56	9.63	6.41	8.61	6.20
	Inservice	20.28	15.63	11.42	19.51	9.98	10.41	12.85
Evaluation: Shelli > Sara	Preservice	42.70	18.42	12.81	1.08	3.45	4.68	16.87
	Inservice	41.68	29.51	8.31	6.32	4.66	3.44	6.08

Next, the two groups' homogeneity was examined with respect to the teachers' ability to notice explicit SRL teaching, and no significant group differences (preservice and inservice) in homogeneity emerged, *F*_(1,601)_ = 0.00, *p* = 0.99, η^2^ = 0.00. Then, to check that the sample met the standards of normality, the asymmetry and kurtosis values were calculated for the participants' noticing of explicit SRL teaching: *M* = 2.66, *SD* = 1.69; asymmetry: 0.33; standard error asymmetry = 0.20; kurtosis = −1.61; standard error kurtosis = 0.40. No deviations from normal values were found based on acceptable asymmetry values of not higher than |2.00| and acceptable kurtosis values outside the interval between |8| and |20| (Bandalos and Finney, 2001). Following these analyses, parametric statistics were used to test the research questions.

Unexpectedly, in contrast to hypothesis 1 predicting better noticing ability among inservice teachers than preservice teachers, no significant differences emerged on the *t*-tests conducted to compare the preservice and inservice groups' mean scores for noticing the videotaped teachers' explicit instruction of planning or evaluation SRL strategies or for the combined Observer score. Only for noticing of explicit SRL monitoring instruction did the two groups differ: The preservice group's noticing scores were significantly lower than those of the inservice group for monitoring; however, as noted above, the inservice teachers' scores did not differ significantly from the neutral score for this strategy. Thus, neither group could notice the SRL monitoring instruction explicitly demonstrated by the expert videotaped teacher (Gila), but the preservice group even erroneously highly endorsed the non-expert teacher in the pair of monitoring-oriented clips (Tali).

Next, to examine the demographic and setting characteristics for the first research question, ANCOVA was conducted for the two teacher groups' ability to notice explicit SRL teaching, with three covariants: teachers' age and sex for the whole sample and school level (elementary/junior/high school) for the active inservice teachers. The analysis yielded no significant differences between preservice and inservice groups' noticing skills as a function of the demographic covariates: age, *F*_(1,601)_ = 1.92, *p* = 0.43, η^2^ = 0.03 and sex, *F*_(1,601)_ = 1.15, *p* = 0.34, η^2^ = 0.06. No significant difference likewise emerged in noticing ability as a function of school level in the inservice group, *F*_(1,305)_ = 4.36, *p* = 0.27, η^2^ = 0.05.

For hypothesis 2 predicting better noticing ability among more veteran inservice teachers, correlations were calculated in the inservice group only between years of teaching experience and noticing of SRL strategy teaching. The findings did not support the hypothesis. As seen in Table 3, the Spearman's rank-order correlation between the combined Observer score and inservice teachers' length of tenure was negative and significant. With regard to the Spearman's correlations between inservice teachers' length of tenure and each of the three SRL strategies, the correlation for noticing of SRL monitoring instruction was negative and significant, while the correlations for noticing of planning and evaluation instruction were not significant although negative in absolute terms. These findings indicated that the more experienced teachers were, in fact, less likely to notice the explicitly demonstrated SRL teaching strategies overall and specifically SRL monitoring compared to their inservice colleagues who had fewer years of experience.

**Table 3:** Spearman rank-order correlations between inservice teachers' years of teaching experience and their noticing of videotaped teachers' explicit instruction of self-regulated learning (SRL) strategies (*N* = 302).

**Noticing of SRL strategy teaching**	**Video clip pair**	**Years of teaching experience**	
	**Expert > non-expert**	***r*_**s**_**	***p***
Planning	David > Natalie	−0.19	0.136
Monitoring	Gila > Tali	−0.27	0.007
Evaluation	Shelli > Sara	−0.17	0.078
Combined score		−0.31	0.002

## Discussion

The current study focused on preservice and inservice teachers' prerequisite first stage of professional vision ability—noticing skills—as uniquely applied here to the domain of explicit SRL teaching in the classroom. Disappointingly, the present results indicated that the majority not only of preservice teachers but even of inservice teachers across their career span failed to accurately identify the videotaped authentic expert teachers' explicit SRL teaching actions performed to directly promote their students' implementation of the three well-established strategies for self-regulation at different phases of learning—planning, monitoring, and evaluation. These preliminary findings in a heretofore neglected area of research suggest that, before even beginning to try to actively implement SRL instruction in their classrooms, many teachers—regardless of their experience levels—may already have difficulty at the initial stage of recognizing the relevant outward manifestations of how to teach each of the three key SRL strategies while filtering out irrelevant teacher behaviors (König et al., 2014).

This apparent deficit in most teachers' basic SRL teaching pedagogical awareness is striking in comparison to prior research findings on teachers' skills for noticing the explicit manifestations of authentic instruction in other pedagogical domains such as classroom management and students' thinking (König and Kramer, 2016; Meschede et al., 2017). The rare prior empirical research on noticing skills specifically directed toward explicit SRL teaching behaviors (Michalsky, 2020a,b) has likewise indicated a difficulty in SRL noticing skills, but these studies could not allow a developmental perspective across the career because they only examined preservice teachers.

The current outcomes uniquely indicated that teachers' ability to notice the instruction of SRL strategies does not seem to reveal the expected progressive improvement coinciding with growing teaching experience. Overall, inservice teachers were no better than preservice teachers at noticing SRL teaching events for any of the planning, monitoring, and evaluation strategies. In fact, unexpectedly, inservice teachers' noticing of explicit behaviors for teaching SRL even appeared to be lower overall among the more experienced teachers, especially regarding how to teach self-monitoring to students. The sources of this negative correlation between noticing skills and years of experience are yet to be explored in a future study, perhaps with a more representative sample of teachers. However, one may speculate that perhaps recent teacher training graduates may have had more direct exposure to SRL pedagogical content knowledge in line with contemporary educational reforms (e.g., Organisation for Economic Co-operation Development, 2014), especially regarding reflection and evaluation, which have become more commonplace in teachers' colleges today than in prior generations. Current reforms in the educational system have raised new goals targeting the professional growth and training of preservice teachers (National Council for Accreditation of Teacher Education, 2014). To this end, many teacher educators are striving to increase preservice teachers' SRL throughout their training period (e.g., Kramarski and Kohen, 2017; Michalsky, 2020b). However, inservice training efforts to inculcate a student-centered SRL teaching approach among more veteran teachers have lagged behind (Karlen et al., 2020).

It is informative to compare the current teachers' skills for noticing SRL to those of university instructors who most likely have already been exposed to the current educational reforms calling for an increased SRL focus. For example, the majority of both preservice and inservice teachers in the current study failed to notice explicit instruction of planning and monitoring strategies—correctly noticed by only 41 and 21% of preservice teachers and by only 39 and 33% of inservice teachers, respectively. In contrast, the majority of university instructors in the study of Perry et al. (2008) did correctly notice these planning and monitoring strategies (62 and 74%, respectively). In the case of SRL evaluation strategy instruction, similarly poor rates of noticing were demonstrated by the current inservice teachers (14%) and by the university instructors in the study of Perry et al. (13%), while the preservice teachers showed a higher rate (25%), although still low. Overall, this comparison suggests that preservice and active inservice K-12 teachers tend to demonstrate less developed skills for noticing teachers' explicit instruction of SRL strategies than university instructors who are not necessarily actively teaching in authentic K-12 classrooms.

## Conclusions and Recommendations

The current unexpected preliminary findings hold implications for methodology and practical application. It is possible that the teachers' inadequate noticing may stem from the current study's “messy classroom” methodology, where the selected videotaped vignettes aimed to reflect the challenges inherent to singling out relevant teaching behavior for contemplation within authentic ill-structured lessons. For example, although a team of expert judges had determined that Tali's clip contained absolutely no explicit behaviors instructing her students to self-monitor, perhaps the current study participants were distracted by Tali's explicit clarification of the written instructions for the students' PISA study task (reading a text accompanied by graphs and answering questions). The teachers may have been unable to differentiate Tali's emphasis on learning the directions for executing the task (e.g., “Remember to check the graph, too”) from learning how to self-regulate their own actions and performance while engaging in the task as appearing in Gila's expert explicit teaching of self-monitoring (e.g., “Check if you understand the graph and, if not, go back to the text”). Another possibility is that the teachers may not have known how to differentiate explicit from implicit teaching, perhaps giving a false-positive score to Tali's implicit arrangement of the learning environment or to her non-verbal behavior. Tali is just one example—there might be others.

Hence, stimuli design should be at the focus of future research to determine the extent to which the current teachers' noticing difficulties may have stemmed from such distractors. In other words, researchers might wish to give the participants clips that present only one strategy at a time for comparison. Another option would be to give teachers a “messy” lesson and ask them to notice as many strategies as possible by naming them and giving their time stamp on the video to enable researchers to check the responses' accuracy. A prior study examining only preservice teachers (Michalsky, 2020b) pinpointed the importance of the type of scaffolds provided to teachers in order for them to notice SRL teaching in videotaped classrooms, demonstrating the best outcomes when preservice teachers were told which SRL teaching strategy was present in the video but not when it would appear and only moderate outcomes when the participants were told when to look (the time range) but not which SRL strategy would appear. Peers who did not receive a scaffold showed poor noticing skills. In the current study, in line with the highest performers in the study of Michalsky (2020b), the participants were told what strategy to seek but not when it would appear in the clip. However, here the teachers needed to compare two clips. Future research should also attempt to validate the current noticing measure by comparing the same teachers' noticing of explicit SRL teaching with their noticing of other pedagogical domains that were previously shown to demonstrate differences between preservice and inservice teachers, such as class climate and lessons' learning goals (Seidel et al., 2001). Such a comparison would indicate if the noticing stage of professional vision is particularly complex and challenging in the SRL teaching domain specifically compared to noticing of other areas.

If so, the apparent deficit in most teachers' basic SRL teaching pedagogical awareness may possibly provide some insights into why even expert, experienced teachers self-report that they rarely teach SRL, as seen on international PISA questionnaires (Organisation for Economic Co-operation Development, 2017). Indeed Dignath and Veenman's (2020) systematic review of 17 studies on observed in-class SRL teaching attempts by diverse teachers with a range of experience levels also previously reported that, in most classrooms, only little direct, explicit strategy instruction is taking place. These results are worrisome. If teachers cannot even identify explicit SRL teaching when it is modeled and demonstrated by experts in authentic classrooms, they cannot be expected to apply such teaching practices during their own instruction, let alone teach students to use such SRL strategies in their independent study. This is unfortunate because Dignath and Veenman (2020), among others, have demonstrated that it is possible to teach students effective SRL strategies by giving them explicit information about such strategies.

The current study also holds implications for preservice preparation and inservice professional development. In light of the considerable difficulty that teachers seem to face in identifying teachers' behavioral repertoire for effective SRL teaching, intervention planners may wish to begin preservice and inservice training in a “clean” environment, without “messy” ill-structured lessons that include multiple SRL strategies. In other words, perhaps the first step in teachers' professional development should be to teach trainees to identify explicit methods for teaching each SRL phase separately (i.e., only planning or only monitoring or only evaluation, without distraction from the other two strategies). Moreover, the present findings imply that professional education should foster teachers' ability to differentiate explicit vs. implicit SRL teaching modes when trying to notice SRL teaching while analyzing real-time classrooms as well as the ability to distinguish SRL teaching behavior from mere task-related instruction behavior. Then, once teacher trainees learn a gamut of ways to explicitly and directly teach each particular strategy (vs. implicit ways for that specific strategy), the training can proceed to help trainees learn about messy environments.

Thus, professional training might do well to systematically and explicitly promote trainees' ability to identify and differentiate the three major SRL strategies from one another and their associated effective explicit vs. implicit teaching behaviors in order to sharpen their understanding. Future research might examine the effectiveness of such training programs for preservice vs. inservice teachers to trace their trajectories of learning and determine if inservice teachers with more experience may be able to capitalize more quickly on these training methods. Moreover, the finding that preservice teachers may currently be more capable of noticing SRL teaching behaviors than their more experienced colleagues, if validated by future research, would call for immediate direct training of the more veteran inservice teachers with regard to SRL strategy teaching. This highlights the vital need for policymakers to urgently promote not only preservice but also inservice professional development.

Drawing conclusions from the current study is limited by its focus on teachers' noticing but not on their actual teaching practices in the classroom. Thus, an important avenue for future research is to examine the extent to which teachers actually do utilize effective planning, monitoring, and evaluation SRL strategies in their real-time classrooms and to investigate the relations between their noticing skills and the strategies that they actually use for teaching. Future research that investigates both noticing skills and actual teaching behavior together in the same sample can verify, for example, whether noticing of relevant teaching/learning events while filtering out irrelevant ones (König et al., 2014) is indeed a prerequisite for teachers' ability to even begin to reason about those events and assimilate them into existing professional knowledge in order to develop pedagogical expertise (Hammerness et al., 2002; Borko et al., 2008; Blomberg et al., 2011; König et al., 2014).

## Data Availability Statement

The raw data supporting the conclusions of this article will be made available by the authors, without undue reservation.

## Ethics Statement

This study was carried out in accordance with the recommendations of the ethics committee of Bar-Ilan University and the ethical principles of the American Psychological Association (APA). Ethics approval was not required as per Bar-Ilan University's guidelines and national guidelines. Inasmuch as the study involved only an anonymous questionnaire and was assumed not to create distress or harm, informed consent was not required according to the APA ethical principles. Nevertheless, participants' consent was obtained as follows: (1) the preservice teachers completed the survey anonymously and voluntarily online in class as a class demonstration; they were informed that the data might also be used for research purposes and were asked to explicitly indicate within the survey whether they consented to their responses being used for that purpose, and (2) the inservice teachers completed the survey remotely, anonymously, and voluntarily online; they were informed that the data was being collected for research purposes by the author; and their consent was obtained by virtue of survey completion.

## Author Contributions

The author conceived, designed, and conducted the study, analyzed the data, and wrote the manuscript.

## Conflict of Interest

The author declares that the research was conducted in the absence of any commercial or financial relationships that could be construed as a potential conflict of interest.

## Figures and Tables

**Appendix 1:**
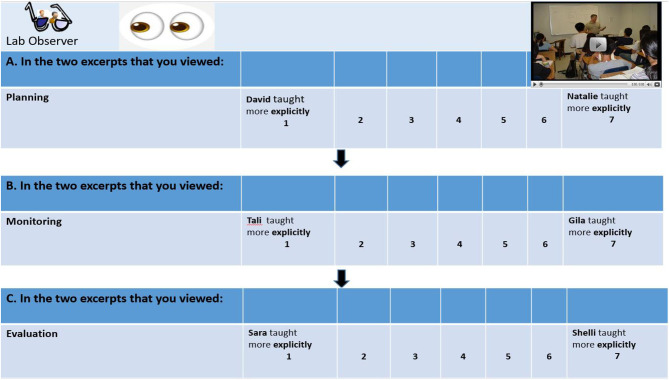
Three screenshot excerpts from the online observer instrument presenting **(A)** the pop-up planning question after viewing the second video clip, **(B)** the pop-up monitoring question, and **(C)** the pop-up evaluation question.
